# 3D Printing of Silk Fibroin for Biomedical Applications

**DOI:** 10.3390/ma12030504

**Published:** 2019-02-06

**Authors:** Qiusheng Wang, Guocong Han, Shuqin Yan, Qiang Zhang

**Affiliations:** Key Laboratory of Textile Fiber & Product (Ministry of Education), School of Textile Science and Engineering, Wuhan Textile University, Wuhan 430200, China; qiusheng-wang@hotmail.com (Q.W.); han792464210@hotmail.com (G.H.)

**Keywords:** silk fibroin, 3D printing, bioink, properties, biomedical applications

## Abstract

Three-dimensional (3D) printing is regarded as a critical technological-evolution in material engineering, especially for customized biomedicine. However, a big challenge that hinders the 3D printing technique applied in biomedical field is applicable bioink. Silk fibroin (SF) is used as a biomaterial for decades due to its remarkable high machinability and good biocompatibility and biodegradability, which provides a possible alternate of bioink for 3D printing. In this review, we summarize the requirements, characteristics and processabilities of SF bioink, in particular, focusing on the printing possibilities and capabilities of bioink. Further, the current achievements of cell-loading SF based bioinks were comprehensively viewed from their physical properties, chemical components, and bioactivities as well. Finally, the emerging issues and prospects of SF based bioink for 3D printing are given. This review provides a reference for the programmable and multiple processes and the further improvement of silk-based biomaterials fabrication by 3D printing.

## 1. Introduction

In recent years, three-dimensional (3D) printing is a promising strategy to the biomedical field and it is regarded as a future alternative to current clinical treatments. Not only that it can alleviate the artificial organ or tissue shortage crisis, but it can also design and produce complex and precise microstructures according to reconstruction of tissue engineering requirements [1,2,3]. More importantly, a series of advanced 3D printing techniques have been approved to achieve structural and functional consistency with model design, which means that competitive manufacturing technology is ready for tissue repair and transplantation [4,5,6]. Bioink as a core of the 3D printing is the key to success for 3D printing products. Specifically, bioinks loading cells, growth factors, and cues for bio-applications are still in the early stage in 3D printing. Therefore, it is an urgent need to seek an appropriate material as bioink for 3D printing.

Bioinks are cell-encapsulating biomaterials that are used in 3D printing process and they must be friendly to both printing process and 3D cell culture [7]. However, most of biomaterials are insufficient in meeting requirements of ideal bioink, so that choosing a suitable biomaterials as bioink plays an significant role in rebuilding the similar function of native tissue following the principle of tissue engineering [8]. In the field of tissue engineering, the three strategies that were used to replace or repair native tissue: using cells, cytokines, or cell substitutes only; using biocompatible biomaterials only to induce tissue regeneration; combination of using cells, cytokines, and biomaterials [9]. Thus, including non-toxic, cytocompatibility, bioactivity, free-standing, and applicable mechanical properties, and cell-loading and encapsulation ability in the physiological conditions, are the pre-requirements and properties of the biomaterial as a bioink. Additionally, when considering the sustainable process of printing, the printability of bioink depends on several controllable parameters, including the viscosity of solution, the ability of crosslinked, and surface tension of the bioink. If the viscosity of the bioink formulation is higher, a larger pressure is needed for the extrusion of bioink from the small nozzle, or causing the nozzle to be blocked and cell death [10,11]. On the other hand, the crosslink mechanism and surface tension are critical to cell’s activity, aggregation, and viability. From the perspective of the biomedical field, time-consuming is a vital factor and can never be ignored, especially in cell-based printing. It usually results a decrease in cell viability for preparation of scaffolds with large and complex structures by 3D printing [12]. The cell-based and cell-free approaches are two categories of bioink used in 3D printing, thus the cell carrier or tissue substitute should keep a balance between self-digestion and tissue regeneration [13,14]. A tunable biodegradability should be taken into consideration, so that the rate of tissue regeneration can be matched. Finally, easy manufacturing or processing that are affordable and readily available are encouraging and welcoming features for selecting suitable biomaterials as bioink formulation [15].

**Figure 1:**
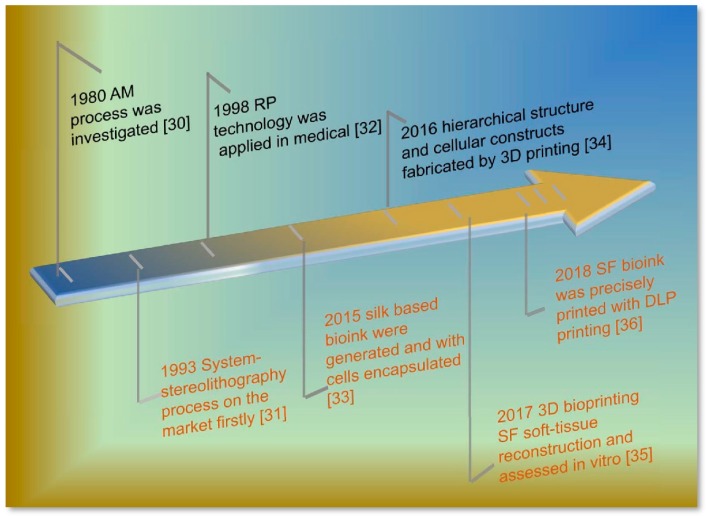
Timeline of the development of Silk fibroin (SF) based bioink in three-dimensional (3D) printing technology [30,31,32,33,34,35,36]. Additive manufacturing (AM); Rapid prototyping (RP); and, Digital light processing (DLP).

Following the rules of ideal bioink, several cases have demonstrated that hydrogels with a high content of water and shape plasticity are attractive candidates as bioinks [16,17,18]. Based on the features, including bio-instructive, cell encapsulation, and a 3D microenvironment, many hydrogels have been developed from naturally derived polymers, such as gelatin, fibrin, collagen, chitosan, alginate, and hyaluronic acid (HA) [19,20,21,22,23]. The gelation mechanism by chemical crosslinking (for gelatin and hyaluronic acid) and ionic (for chitosan and alginate) are not suitable for the bioactivity of cell-loading bioink, and the inappropriate degradation rate (for fibrin and collagen) also shows an unfavorable servicing. Previously, a series of Silk fibroin (SF) products gained much attention for application and they were studied as a protein polymer for biomedical applications, for instance, in the enzyme immobilization matrix [24], wound dressing [25], vascular prosthesis [26], and artificial grafts [27], due to its similar components to the extracellular matrix (ECM), low-cost, tunable mechanical properties, controllable degradation, and good biocompatibility [28,29]. The timeline of the development of SF based bioink in 3D printing technology [30,31,32,33,34,35,36] over the past 30 years has witnessed great research and application value for the customized biomedical filed (Figure 1). These results encouraged further exploration of the SF based biomaterials via 3D printing. 

In this review, we firstly discuss the evolution toward 3D printing derived from SF (*Bombyx mori* silkworm) bioink, mainly focusing on the improvement and design of SF bioink to match the requirements of ideal bioink. Subsequently, we summarize the advanced progress in biomedical applications that are based on 3D printing of SF bioink in vitro. Finally, we outlook the broader challenges and directions for the future development of SF bioink for functional materials designs and engineering via 3D printing.

## 2. Silk Fibroin Bioink

### 2.1. Processing of SF Bioink

Native *B. mori* silk is composed of silk fibroin protein coated with sericin protein, and sericin is a group of soluble glycoproteins that are expressed in the middle silk gland of *B. mori* silkworms [16]. By degumming, the sericin is removed, the SF fibers could be dissolved and purified into an aqueous solution through dialyzing in deionized water [37]. Based on aqueous solution system, the SF can be further processed into different types of materials in films, particles, fibers, and sponges, also including hydrogel. However, there is a barrier hindering 3D printing fabrication in SF bioink that is caused by low concentration and viscosity. Increasing its concentration and adding other high viscosity additives are perhaps useful strategies in improving its printing processability and biofunction ability.

**Figure 2:**
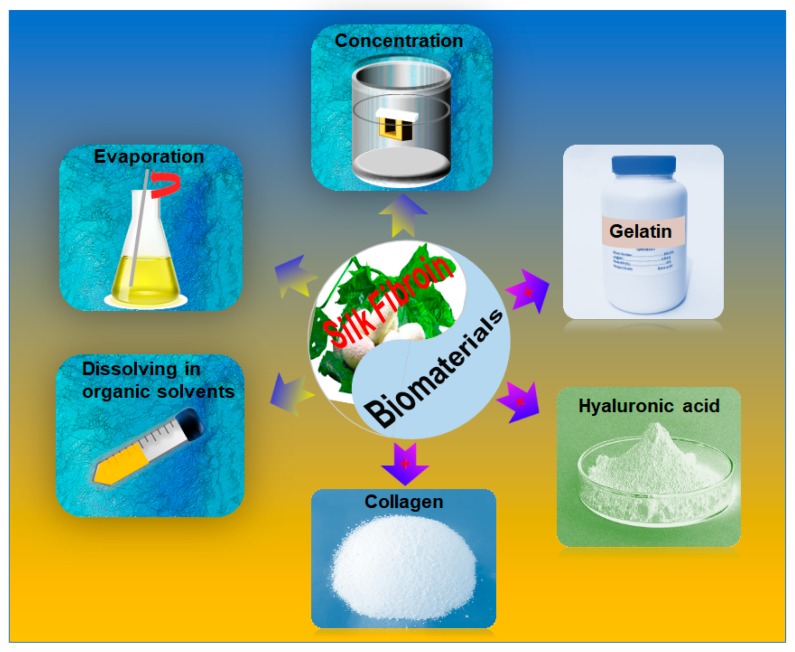
Schematic of methods to optimizing the rheology of SF bioink. SF is a biomaterial with impressive biocompatibility and mechanical properties. As a bioink, its rheology should be adjusted in aqueous system by different strategies. The gradient arrow without “+” indicated that their rheology could be regulated by concentration, evaporation, and dissolving in organic solvents; the arrow with “+” shows that SF solutions were combined with other biopolymers, such as collagen, hyaluronic acid, and gelatin, respectively, to enhance their rheology.

To obtain high concentration SF solution, as shown in Figure 2, there are two approaches that are employed. One way is based on the SF purification protocol that is modified with some additional procedures. Specifically, SF solution is concentrated with a dialysis bag (Molecular Weight Cut Off (MWCO) ≈ 3000 Da) in polyethylene glycol (PEG, Molecular Weight (MW) ≥ 20000 Da) solution, or regenerated SF materials are re-dissolved in organic solvents (1,1,1,3,3,3-hexafluoro-2-propanol (HFIP), Formic acid, etc.) to increase the concentration to meet the requirements of rheology of bioink [17,18]. However, the bioactivity of silk proteins will be inevitably weakened by these complexing processes. Recently, adapting new dissolving systems for another effective way, the Ca^2+^-formic acid binary solvent system and HFIP are studied as dissolving solvent directly for silk fibers to produce high concentration SF solution [38,39], which is easier for yielding over 20 wt.%. These unfriendly solvents will continue cutting the SF molecules chains in a further process, resulting in low SF molecular weight and viscosity. What is more, the unfriendly solvent residues have a detrimental effect on cell viability and encapsulating in 3D printing, which limited the applications of these solvents in 3D printing. As a second strategy, it is convenient and highly efficient to enhance the free-standing and viscosity of SF based bioink by blending other high viscosity biomaterials. Based on the principle of similar compatible, gelatin, chitosan, alginate, and HA are mixed with SF solution to prepare SF based bioink [33,36,40]. This strategy is more successful than other approaches in improving the SF solution with a high concentration and plastic ability for 3D printing.

### 2.2. SF Bioink Design

Nowadays, although synthetic polymers broaden the diversity of materials, their low cell viability and non-biocompatible degradation products hinder making a further step as bioinks. Natural materials, like cellulose, HA, and collagen, are friendly to cell growth and development as SF materials, while the slow gelation rate or inappropriate mechanical properties always mismatch with rapid additive manufacturing technology [41,42]. Encouraging by the easy processing and abundant source, SF, as a bioink, motivated more researchers to explore their wide range of applications.

**Figure 3:**
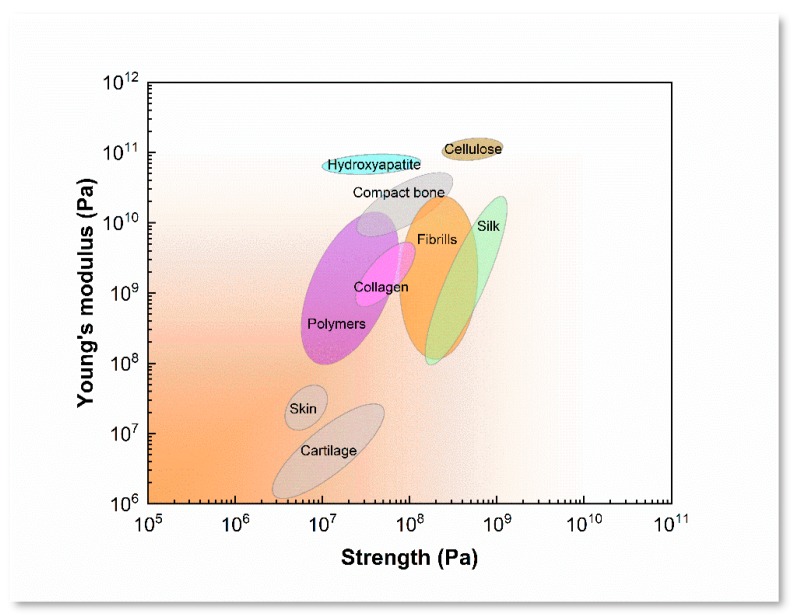
Comparation of the specific values of strength and stiffness of SF materials with natural and synthetic materials. Reproduced with permission from [65]. Copyright 2011, Nature.

By contrasting with the characteristics of SF and polymers that are mentioned above (Table 1), single-SF is probable to yield into bioink for 3D printing in aqueous system. According to the LiBr-dissolving protocols, SF bioink is treated to optimize its rheological ability via the purification and concentration process by slowly stirring and low temperature evaporation, and their mechanical properties and degradation could be controlled by the regulation of β-sheet content, degree of crosslinking, and morphological structures [43,44]. Nature silks have showed a lot of features, such as outstanding strength and toughness, controllable degradation, and high cell viability (Figure 3). The regenerative SF materials usually resulted in the deterioration of mechanical properties, which could be reinforced by inducing conformation transition. Specifically, several approaches are employed for transformation random coil or helical conformation into β-sheet structure to induce the SF insoluble, such as alcohol solution treatment [45], soft-freezing treatment [46], shear force inducing [47], salts addition and crosslinker [48], and pH value adjustment [49]. These approaches may be used to enhance the free-standing of SF 3D printing scaffolds and regulate their biodegradation in vitro and in vivo. These characteristics also indicated that the printability and mechanism of SF bioink could be controlled to meet different printing purposes.

When considering that function of biomaterials in the reconstruction of neo-tissue by providing a stable and biocompatible microenvironment for cells proliferation and differentiation in tissue engineering [62], the bioink should be designed intensively. SF is one of the most studied and industrially used types of fibrous proteins in biomedical applications. Several attempts have been made in biomedical with 3D printing technology. However, some aspects of SF bioink should be addressed based on previous cases. Specifically, from the point of a physic-chemical view, the printability of bioink should take care of some parameters, including rheology, swelling ratio, and surface tension [14]. First, the excellent rheology is the basic requirement for bioink that was extruded from the nozzle, as the higher extruded-forces would harm cell viability [63]. The proper swelling ratio is beneficial to the formation of certain two-dimensional (2D) morphological structure after the bioink extruded, which have a role in improving resolution and free-standing of printing products. Third, more attention should be paid to surface tension, which exists between the compounds that are present in the liquid. It plays a big role in building a 3D structure for cell attachment distribution and development [64]. The surface tension should be self-adjustable so as to meet the changes that the surface tension imposes on the liquid-gas interface [14]. Moreover, from a bio-fabrication point of view, the excellent cell-encapsulating or growth factors-loading abilities are significant for cell proliferation and adhesion. Hence, the SF bioink based on aqueous system or cell culture medium system should put more efforts into retaining them in future studies.

**Figure 4:**
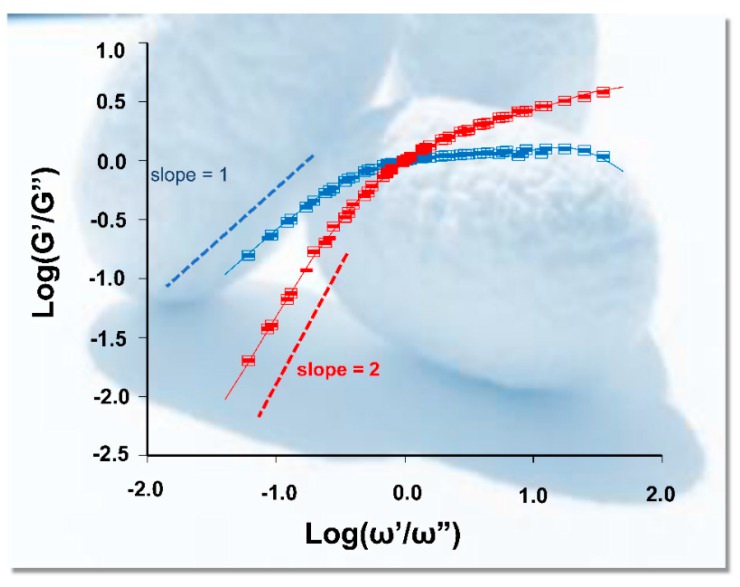
The relationship between loss factor and frequencies of SF. Reproduced with permission from [69]. Copyright 2016, American Chemical Society.

Regarding the bio-inspiration of silkworm spinning, the process of silk cocoons formation is a typical procedure of architecture by the 3D printing technique. There is no doubt that silk protein solution is an ideal and attractive choice for bioink [40]. Because of the existence of sericin, silks are easy to spin and build into the silk cocoons approach to 3D printing by silkworm. The natural behavior of silkworms highlights to us that single component SF is insufficient for 3D printing. Blending and hybrid bioink should be considered in improving in the aspects of printability, especially for rheology and viscosity [51,66]. Wet spinning or microfluidic spinning cases demonstrated that the two factors for rheology and viscosity of fluid included deformation energy stored (G′) and dissipated energy (G″) [67,68]. As shown in Figure 4, the SF G′ always exceeded G″ at high frequencies and vice versa at low frequencies, which means that it is conductive as viscoelastic liquid, and these characteristics determine the rheology of multicomponent bioink [69].

The basic physical characteristics of SF bioink should not only be addressed, but some chemical characteristics are also helpful in optimizing its printing abilities, especially in self-assembly [70], chemical decorative [71], and conformation transition induction. On one hand, once the amino acid sequence of SF self-assembled into an antiparallel β-sheet structure by intra- and inter-molecular hydrogen bonds [72], which would contribute to robust mechanical properties. On the other hand, the presence of several reactive amino acids in SF allow for easily accessible chemical modification strategies, including coupling reactions [73], amino acid modification [74], and grafting reactions [75]. Based on chemically modifiable of SF, the recently report showed that SF could be modified with methacrylate groups directly for light polymerization, which would be beneficial to improve its printability [36]. These strategies are utilized to tailor the protein for a desired function or form [76]. Based on physical and chemical characteristics of SF solution, SF bioink shows a strong vitality for 3D printing when it combined with other biomaterials via optimizing the basic parameters of the bioink, such as printability, mechanical properties, shape fidelity, and cell viability [77] (Table 2). Raw material screening and formula optimization usually are the initial and essential steps in multicomponent bioink. As mentioned before, the combination of SF with polysaccharide bioink is an effective approach to adjust rheological properties, such as chitosan, alginate, and HA. The gelation rate and printability can be improved significantly with alginate being applied as an additive component [78]. Gelatin as another great candidate for modulating SF based bioink properties gains much attention due to its similarity to human ECM and with a gentle gel environment, and its rapid degradation rate and weak mechanical properties are supported by the incorporation of SF [79]. Therefore, it will promote 3D printing technology to develop a SF based-multicomponent bioink to overcome the shortages of single bioink.

Besides the basic physical and chemical characteristics of SF bioink, the biological performance is another essential indicator that can never be ignored in bioinks. Over past decades, numerous studies witnessed and proved the excellent biological properties of SF, and properly degummed and sterilized silk manufactures demonstrated biocompatibility and bio-viability that were as good as commercial products of polylactic acid and collagen [80]. The United States (U.S.) Food and Drug Administration approve of these products. SF bioink performances are described as followed in: (1) huge cell-loading printability for precisely control SF ink deposition [81], which has advantages in overcoming the uncontrollable cell dynamics [82]; the mismatch of printing process parameters [83]; (2) the good encapsulation ability for cells, drugs, and bioactive molecules [84,85]; and, (3) excellent viability for different cells and cell lines for proliferation and differentiation [86].

However, as shown in Table 2, choosing a suitable 3D printing technology and method is also the key to the success of SF bioink. Firstly, the concentration and rheology of SF should match the requirements of different additive manufacturing technology. For instance, the low viscosities (3.5–12 mPas^−1^) behave in inkjet printing better than extrude printing (about 600 kPas^−1^) [91,92], and the large mechanical stress that is applied to extrude the bioink resulting in the reduction of cell survival. Secondly, adapting controllable physical gelation or phase transition strategies so that the toxicity of crosslinkers to cells is reduced and cell encapsulation is enhanced [93]. Take the photo-crosslink as an example; it is an effective method improving the cell viability for the chemically modified SF [36]. Thirdly, the printing resolution of SF bioink is susceptible to printing parameters, such as temperature, printing speed, and SF molecular weight. As for as the resolution of 3D printing, laser-based printing have a high resolution of 1–3 μm, but it has to solve cell-damage that is induced by laser [94]. Extrude printing technology with a low resolution (about 100 μm), which was applied more in the most recent researches [95]. Additionally, a great potential for inkjet printing is attached to more future study for its relative high resolution of 50 μm and high printing speed [96].

## 3. Evaluation of Cell Viability with SF Based 3D Printing Scaffolds

**Figure 5:**
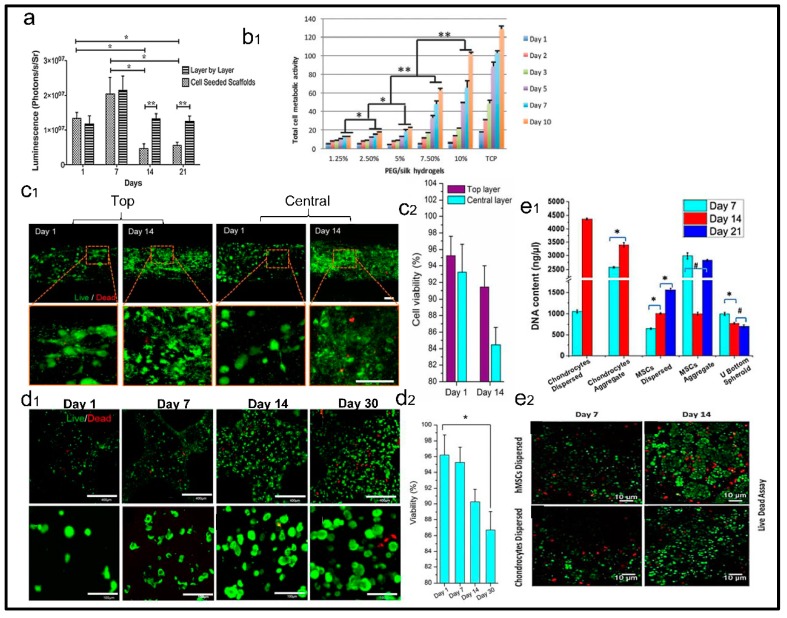
The evaluation of cells with printing bio-model in vitro. (**a**) the cells number assessment of lay-by-layer (LBL) sandwiches and the cell-seeded scaffolds (CSS). Reproduced with permission from [100]. Copyright 2012, Mary Ann Liebert, Inc. (**b**) Total cell metabolic activities with different concentration of SF. Reproduced with permission from [97]. Copyright 2018, Wiley. (**c**1,**c**2) Cell viability within the decellularized adipose tissue (DAT) constructs in top and layers. Reproduced with permission from [101]. Copyright 2015, Elsevier. (**d**1,**d**2) Live/Dead images and quantitative analysis of human turbinate mesenchymal stem cells (hTMSCs) that were encapsulated in tyrosinase crosslinked 8SF-15G-T constructs over 30 days. Reproduced with permission from [50]. Copyright 2014, Elsevier. (**e**1,**e**2) Cell viability and proliferation. Live and dead cell assay of hMSCs and chondrocytes printed with silk-gelatin bioink as dispersed cell at three weeks. Reproduced with permission from [40]. Copyright 2016, American Chemical Society.

As a bioink, cell viability is another key point of its success in 3D printing. There are several cases regarding 3D printing creations that are based on SF bioink [35,36,50,97,98,99]. 3D printing artifacts allowed for cell seeding that is more efficient than that of porous scaffolds derived from freeze-drying and electrospinning techniques [100] (Figure 5a); their precise mimic nature tissue framework could regulate cell phenotypes and neo-tissue reconstruction by stimulating cell differentiation and proliferation. The component of SF also acted a positive influence on the biocompatibility and bioactivity of 3D printing scaffolds via providing a different stiffness and rough morphology [97] (Figure 5b). In fact, the microenvironment and time-consumption of printing objects should match with the cell aggregation and proliferation disciplines. In order to maintain long-term cellular viability, desired cellular distribution and mild mechanical action are necessary during 3D printing. Previous studies show that the cell viability of top layers is better than that of central layers after 14 days, which may be ascribed to a 3D open-porous structure that facilitated, at a certain extent, the diffusion of nutrient and oxygen to the encapsulated cells during their residence. In addition, the layer by layer manufacturing technology is a time-consuming process that is influenced by the cell fate of the cell-loading bioink directly [101] (Figure 5c1,2). Secondly, a friendly or low side effective crosslinking method should be adopted in improving cell viability. For instance, the cell viability shows a trend of significant decrease for SF-alginate bioink with crosslinked tyrosinase. The results showed that the tyrosinase-crosslinking has an unfavorable effect on cells encapsulation in the long term (30 days) [50] (Figure 5d1,2). The physical crosslinking method may be a proper approach for 3D printing fabrication. Thirdly, the equal environment should be adapted by multi-cell to predict whether the printed object acts upon an implant, causing immune rejection in the body or not. As shown in Figure 5, the chondrocytes and human mesenchymal stem cells (hMSCs) were cultured on 3D printed silk-gelatin scaffold, respectively, the cellular dispersion increased significantly both kinds cells, but the cellular aggregate changed toward opposite directions [40] (Figure 5e1,2). Finally, the bioactivity and mechanical performance are insufficient at the initial stage or after implantation for a while, which usually caused a cavity or cyst in defect sites by supporting deficiency. Consequently, these primary results inspired us with courage in understanding the biological mechanisms of cells and the fabrication of biomedical materials.

## 4. SF Bioink for Biomedical Applications

### 4.1. Skin Tissue

**Figure 6:**
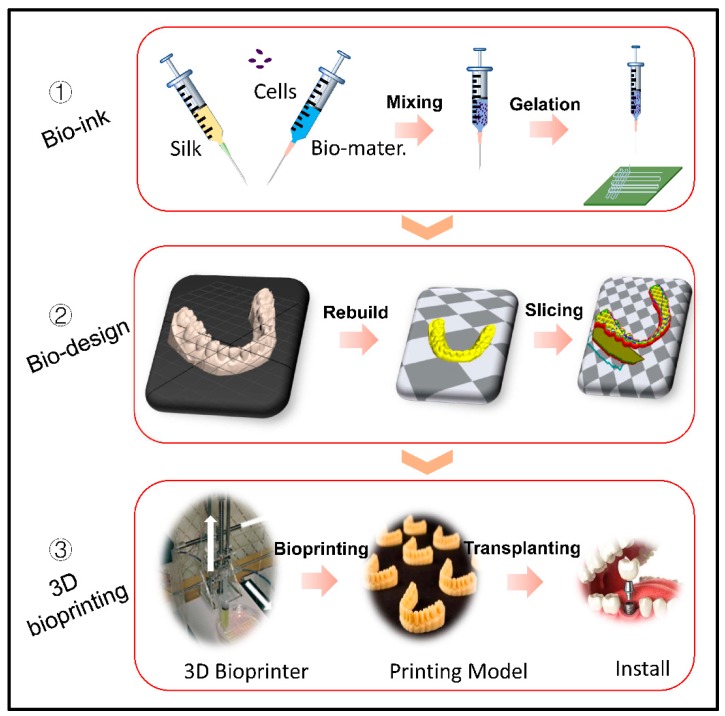
Schematic of 3D printing process. (1) Preparation of SF based bioink for 3D printing (2) Imaging and digital design. (2) 3D printing process and clinical trial. Reproduced with permission from [97]. Copyright 2018, Wiley.

With the development of multicomponent bioink and printing technology, a series of biomedical applications have been reported based on the process of 3D printing (Figure 6). The skin is the largest complex organ in the human body and it is composed by three layers (the stratified squamous epithelium, the dermis, and the hypodermis) [102]. Autografts and allografts are two strategies for skin repairing, which is still limited in donors and recipients to some extent. Specifically, the donor suffers from pain, second operation, and scarring, for the recipient, with the exception of scarring, dermal vascularization, and epidermis functionalization, are difficulties facing their subsequent therapy [103]. Recently, a gelatin-sulfonated silk composite scaffold was fabricated by a DIY pneumatic printing system, with the incorporation of growth factors, which presented skin-like tissues and enhanced skin regeneration by printing technology [79]. By the nanoimprint lithography technique, SF film with skin tissue-like nanoscale structures was fabricated to mimic the collagen morphology of natural dermal [29], which is known, as it could alleviate scar formation. The silk-based bioink combined with collagen are also employed to prepare artificial skin grafts, and the network connective of neo-tissue increased alot when compared with scaffolds that are derived from the freeze-drying method [104]. Although SF as bioink to printing artificial skin-tissue is starting out, the available results regarding the histology and immune fluorescence characterization of the 3D printed grafts presented an applicable potential in skin tissue repair.

### 4.2. Cartilage Tissue

Cartilage damage and degeneration are common disease in the aged suffering from osteoarthritis, which has become an urgent need in clinical healthcare [105]. Some challenges still existed in mimicking the fine structures of native cartilage tissue, especially in nano- micro-ordered structures. Fortunately, when comparing to common approaches, the 3D printing fabrication manifested positive practicability [106]. It appeared to be more promising for SF based bioinks with the recent study, though it was not wildly applied in tissue engineering [107]. For example, by integrating SF with gelatin loading growth factors as bioink, it could be optimized in structural and function for cartilage repairing [40,87]. Pure silk bioink with high concentration could be processed by direct-writing technology, which showed that 3D printing is a much more competitive method in resolution, cell viability, and complex tissue formation [108].

### 4.3. Bone Tissue

Bone tissue engineering usually relies on bone structure, compositions, mechanics, and tissue formation, which makes it crucial in obtaining a fundamental understanding of bone biology [109]. Nevertheless, it has become the focus as to how to keep balance between bioactivity and mechanical properties for printing bone [110]. As for mechanical performance, the bio-ceramics have been used frequently as an important element of bioink, including α-tricalcium phosphate (α-TCP) and hydroxyapatite [61,111,112]. The results showed great potential in bone tissue repair when combined with SF. For instance, polylactic acid/hydroxyapatite/silk ternary bioink, in fabricating bone clip, which demonstrated an equivalent mechanical property, good biocompatibility, and alignment when compared with other types of the bone clip [113]. Another SF/hydroxyapatite scaffold that was fabricated by direct-writing technology and their regular pore size was beneficial regarding cell growth in orientation [29]. A low-temperature printing technology for the collagen/decellularized extracellular/SF scaffold preparation also showed higher cell proliferation and differentiation. When comparing to that of the collagen scaffold, the compressive modulus was highly improved due to the β-sheet formation of SF [114].

### 4.4. Blood Vessel

Vasculature within the tissues or organs is crucial in transporting oxygen and nutrients and in maintaining tissue functions [115]. Though the quantity demanded is enormous, the thrombogenicity and low patency rate narrowed the clinic utility of artificial blood vessels, especially in repairing small diameter (in 4–6 mm) blood vessels [113]. By convenience of 3D printing, it was greatly encouraged to manufacture blood vessel tissue engineering. SF and glycidyl methacrylate (Sil-MA) as blending bioink was used for building blood vessels in the hydrogel state; the hydrogel showed outstanding mechanical and rheological properties, which provide many possibilities for vessels, brain, and ear with highly complex organ structures [36]. Similarly, the SF incorporated melanin nanoparticles could be as a transparency modifier to adjust poly(ethylene glycol)-tetra acrylate to improve the printing resolution, and these features make it possible to fabricate blood vessels or vacant tubes [116]. In advanced 3D printing technology for fabricating vessels, preference should be given to obtaining enough porosity and mechanical properties and non-thrombosis to combat thrombogenicity at early stage [117]. Therefore, the characteristics of SF bioink should be optimized to satisfy target application and tissue engineering [118].

## 5. Summary and Prospects

3D printing has become a promising technology and it has gained high and extensive attentions in silk biomaterials. SF, as a natural and ancient protein material, was a great promise candidate for bioink. In this review, we summarized the physical, chemical, and biological characteristics of SF, and deliberated the proceeding methods and contemporary issues of SF as bioink. Although many cases of SF based bioinks have been reported continuously, taking this technology from the bench to the bedside still requires focused efforts on many fronts.

Firstly, because most of the established methods are time-consuming and require a series of chemical reagents, these options can undermine the green and biocompatible features of biomaterials. 

Secondly, as a bioink, SF should be designed and processed synthetically, especially in its viscosity, rheology, encapsulation, and biocompatibility. The potential approaches are the concentration of the SF solutions and the incorporation of other biopolymers. With the aim of tissue reconstruction, the various cells and growth factors are highly recommended during 3D printing. A combination of these biopolymers in silk materials can compensate for the limitations of individual components. These have potential to enhance the performance and function of the final materials by 3D printing. 

Finally, the homogeneously distribution of the cell before and after printing in bioink is an important parameter to be controlled. From the perspective of manufacturing technology, only by choosing the biomaterials and finding a suitable cell-seeding method can this trouble be resolved. At the same time, optimizing the biodegradation rate of the SF creations to match the speed of neo-tissue regeneration is necessary. 

In summary, we established an overall view in understanding the requirements in 3D printing of the SF bioink. The fundamental understanding of this biological ink can accelerate the development of new methods to obtain novel 3D biomaterials and it offers the opportunity for regarding insight protein material designs in biomedical applications.

## Figures and Tables

**Table 1:** Comparative analysis of silk versus other pure polymeric bioinks.

Materials	Advantages	Disadvantages	Crosslinking Methods
Silkworm silk	Ease of structure modification [37] Controlled degradation High cellular viability Diversity of methods for crosslink or sol-to-gel [50] Outstanding strength and toughness Embedded hydration properties [28] Abundant sources	Rheology need to be optimized as bioink [51] Low viscosity [52] Hard to printing individually	Enzymatically Temperature pH value changes Sonication Salting leaching Photo-crosslink
Alginate	Ease of crosslinking Stability of constructs Biocompatible, facilitates cell entrapment Ease of processability [53]	Fast degradation in vitro, need additional dopants Low cell attachment and protein adsorption Lack of adequate mechanical properties	Ionic (Ca^2+^)
Agarose	Non/low-toxic Biological properties can be improved with other hydrogel easily Suitable mechanical properties for cartilage tissue repairing [54]	Non-degradable Not suitable for inject printing with high viscosity Low cell adhesion and spreading	Low temperature
Collagen	Easy degradation Facilitate cell adhesion and cell attachment Easy to modify with other polymers Need to improve its mechanical and biological properties with other polymers [41]	Time-consuming for gelation Complex process to purification Low mechanical properties Biorisk	pH Temperature Vitamin Riboflavin Tannic acid [55]
Fibrin	Excellent biocompatibility and biodegradation [56] Rapid gelation Easily purified from blood providing autologous source Superior elasticity	Weak mechanical properties Severe immunogenic responses So fast for its degradation	Enzymatic treatment
Cellulose	High mechanical properties Helpful for improving cells viability [57] Excellent shape fidelity [58]	Environment sensitive Non-biodegradation in vivo Purification	Ca^2+^
Hyaluronic acid	Fast gelation Controllable mechanics, architecture, and degradation Supports cell adhesion, migration, proliferation [59]	Weak mechanical properties Need chemical modification to regulate the rheology.	Photo-crosslink
Hydroxyapatite	Keep good shape fidelity and produce porous [60]	Slow degradation rates [61] Low bioactivity	Methanol

**Table 2:** The properties of bioink formulated by multicomponent materials based on SF.

Bioink Formulation	Crosslink Method(gelation)	Cell Types & Density & Viability	Advantages (A) and Disadvantages (D)	Applications	Printing Method	Ref.
SF-Gelatin	Enzymatic/sonication	hTMSCs; BMSC 2.5 × 10^6^ mL^−1^; 2 × 10^5^ 86% (30 days); enriched (21 days);	A: Enhances cell adhesion Good mechanical	Artificial Implant/Cartilage tissue engineering	Inject printing	[50,79,87]
SF-Collagen	Ethanol	BMSCs 2 × 10^7^ cells 4 × 10^2^ cell (13 days);	A: Comprehensive physical properties; support cell growth	Knee cartilage; Tissue engineering	Extrude printing	[88]
SF-Chitosan	hexamethylene diisocyanate/chlorohydrin/glutaraldehyde	BMSCs 2 × 10^7^ mL^−1^ 10^2^ cells;	A: Produce high porosity with different structures; D: the cross-linking agent have cytotoxic	Tissue engineering Drug release	Extrude printing	[88]
Cartilage acellular matrix (CAM)-SF	Enzyme (EDC-NHS)	rBM-MSCs Seeding efficiency 65% >80%;	D: Poor shape fidelity; low precision of printing	Cartilage tissue engineering	Extrude printing	[89]
SF-Alginate	Horseradish peroxidase (HRP)-H_2_O_2_	NIH3T3 5 × 10^5^ mL^−1^ begin to decline slowly (42 days);	A: maintain long-term metabolic activity for bioink D: the compatibility of silk and alginate need to be improved.	Vascular tissue engineering	Inject printing	[78]
SF/polyethylene glycol (PEG)	Sonication	hMSCs 2.5 × 10^6^ mL^−1^ 50% (3 weeks);	A: maintain shape for a long time (6weeks); the crosslinker without damage cell viability; with a good mechanical and high shape fidelity	Cartilage tissue engineering	Inject printing	[90]
SF-glycidyl methacrylate	Photo-crosslink	NIH/3T3 1 × 10^6^ mL^−1^ 50% (4 weeks)	A: a gentle crosslink environment and friendly to cells growth; the mechanical properties improved with Sil-MA concentration increased.	Bone tissue engineering	Digital light printing	[36]
